# Enhancing Technical Performance of PVC Production: A WEP-Based Energy and Water Assessment

**DOI:** 10.3390/polym17111561

**Published:** 2025-06-04

**Authors:** Rolando Manuel Guardo-Ruiz, Linda Mychell Puello-Castellón, Rodrigo Ortega-Toro, Eduardo Andrés Aguilar-Vásquez, Ángel Darío González-Delgado

**Affiliations:** 1 Nanomaterials and Computer Aided Process Engineering Research Group (NIPAC), Chemical Engineering Department, Universidad de Cartagena, Cartagena 130015, Bolivar, Colombia; rguardor@unicartagena.edu.co (R.M.G.-R.); lpuelloc2@unicartagena.edu.co (L.M.P.-C.); eaguilarv@unicartagena.edu.co (E.A.A.-V.); 2 Food Packaging and Shelf-Life Research Group (FP&SL), Food Engineering Department, Universidad de Cartagena, Cartagena 130015, Bolivar, Colombia; rortegap1@unicartagena.edu.co

**Keywords:** water integration, water–energy–product analysis, responsible production, direct recycling, PVC, computer-aided process engineering

## Abstract

Polyvinyl chloride (PVC) is one of the most widely used polymers due to its physical properties and versatility. Water consumption of the suspension method is a critical issue that hinders competitiveness. In that case, this study implements water integration through direct recycling, with the aim of minimizing both freshwater consumption and wastewater generation. The source–sink diagram was used to generate the recycled water network, and the integrated process was simulated using software. From simulation data, the water–energy–product (WEP) analysis method was used to assess the process performance, and sustainability indicators for water, energy, and product were evaluated. Fractional water consumption and wastewater production ratio indicators increased to 51.1% and 55.0%, compared to 41% and 54% in the non-integrated process, showing improved water efficiency and cost reduction. The unreacted material reuse index reached 100%, while the production yield was 99.8%, due to effective recycling of unreacted VCM. The use of natural gas and energy integration led to optimal performance in TCE, NGCI, and EECI indicators. However, the ESI indicator was high (3.59 MJ/t) due to energy demands from thermal control equipment for water recirculation.

## 1. Introduction

Polymers are currently considered one of the most important materials for society. These kinds of synthetic materials present different physical properties that allow their implementation in different productive areas, such as good chemical resistance, lightness, and durability [1]. One of the most widely used polymers due to its properties is polyvinyl chloride, among which are resistance to abrasion and impact, lightness, durability, and impermeability. At the industrial scale, the production of polymers is on the rise due to the increase in the use of these compounds. The production of plastics has dramatically increased since the mid-1960s. World production has increased twenty-fold since that decade, with a doubling of this increase expected by the 2030s [2].

Due to its wide range of applications, it is necessary to constantly improve the production process to take advantage of the raw materials used to obtain it. About 80% of the world production of PVC is carried out by suspension polymerization, using monochloride (VCM), stabilizers, and an initiator in an aqueous medium (water) [3]. Suspension polymerization allows greater control of operations and favorable economic profitability compared to other processes such as emulsion polymerization [4]. At the same time, it allows for achieving a high percentage of productivity and flexibility in the handling of the polymeric composition.

Computer-aided process engineering (CAPE) is based on the development of different systems oriented to the generation of products and processes, based on different aspects such as the phenomena involved or the scale of production. CAPE process engineering employs modeling, design, optimization, and problem solving to improve the production and restructuring of systems, which directly impacts their efficiency and performance [5]. Suspension polymerization of PVC is a complex process, governed by multiple reaction principles that include the interaction between the compounds (phases present) and the resulting polymer properties [6]. To understand and optimize this process, it is essential to evaluate aspects such as the kinetics and thermodynamics of the reaction. Bárkányi et al. [7] developed specific methodologies to address these aspects, laying the groundwork for detailed modeling and simulation of the process, as evidenced in subsequent studies.

Modeling and simulation of the suspension polymerization process of PVC have been the subject of study and implementation throughout various investigations, with the objective of optimizing and better understanding this industrial process. Silva et al. [8] developed a modeling approach for the polymerization of PVC in suspension, focusing on reaction kinetics and operating parameters, with the objective of simulating the dynamic behavior of industrial batch reactors. Their model allows accurate prediction of polymer properties and supports control and optimization efforts. Kiparissides et al. [9] provided a prediction of relevant aspects in polymerization reaction, calculating the viscosity, morphology, and agitation power in suspension polymerization of PVC, thus facilitating the analysis and optimization of the process on an industrial scale. Simulating the reaction dynamics, in order to replicate the real process behavior, has also been an area of interest [10].

Thermal management of the polymerization reactor is crucial to prevent issues such as runaway reactions or suboptimal polymer properties [11]. Wieme et al. [12] developed a model to evaluate the reactor cooling with the implementation of cooling jackets, integrating it with the suspension polymerization model and using heat balances for reaction prediction [13].

Aguilar-Vásquez et al. [14] extended these efforts by developing a complete simulation of PVC production using suspension polymerization in AspenPlus V14, designing a production plant of 1158 tons per day.

It is important to note that PVC polymerization presents significant environmental challenges, such as high energy consumption, emission of toxic substances, smog generation, and intensive water use [15]. In response to these challenges, strategies such as water recirculation have been proposed, with the objective of reducing wastewater generation, reducing freshwater consumption, and, consequently, improving the sustainability and costs of the process.

The intensive use of water in the PVC process allows the application of process integration techniques, such as water integration. Water integration is a holistic and systematic approach that identifies achievable performance targets to improve water conservation [16]. This is achieved by optimizing the allocation, separation, and generation of flows and species based on a fundamental understanding of the overall water flow within the process [17]. There are several methods for implementing water integration, which include graphical and mathematical techniques [18]. Among these, direct recycling and reuse networks are the most widely applied, as they offer significant benefits in water utilization with minimal economic costs [19].

Chan et al. [20] integrated a PVC process and found a wastewater reduction of 90.1% and freshwater minimization of 31.7%; it should be noted that these savings were achieved by effluent storage (indirect recycling). However, Chan’s study focused on the optimization of the polymerization reaction without addressing its implementation in an industrial plant or considering the equipment involved and the necessary operating conditions.

On the other hand, limitations arise due to the composition of the process effluents, as noted by Zheng et al., who found similar results to Chan et al., with a higher reduction in wastewater (48%) than the savings in freshwater (26%) when integrating a plant that produces sodium hydroxide and PVC. The composition of the process wastewater significantly limited effluent recycling due to its composition (organic compounds) [21]. Additionally, potential savings are only analyzed from a purely technical–economic perspective, such as Lee et al., who combined production scheduling and water integration by linear programming (GAMS) to minimize water consumption (flow and cost). A reduction of 32,720 tons per year of freshwater was obtained with a minimum cost of 65,267 per year. Additionally, a 52,667-ton storage tank is required to store wastewater at 50 ppm [22].

While previous studies have individually focused on modeling the polymerization process, evaluating operating conditions, or integrating water reuse strategies, few have addressed the intersection of these aspects from a unified resource-efficiency position. In particular, most previous works have omitted a detailed treatment of plant-wide operating conditions and equipment or have limited their analyses to technical or economic parameters without a comprehensive view of material and energy use. The novelty of this study resides in the overall integration of water reuse strategies—specifically through direct water recycling—within a full-scale PVC suspension polymerization process, evaluated through a systematic assessment of water, energy, and product usage. By combining dynamic process simulation with the water–energy–product (WEP) methodology, this work introduces a consistent and quantifiable framework for assessing process performance in terms of resource efficiency [23]. Unlike previous studies, this approach not only simulates the full-scale operation of the plant but also quantifies the consumption and distribution of key resources, providing a robust and structured basis for decision-making. Thus, this research offers a novel contribution to the optimization of PVC production, addressing a critical methodological gap through the integrated evaluation of resource use within the process.

## 2. Materials and Methods

**Figure 1:**
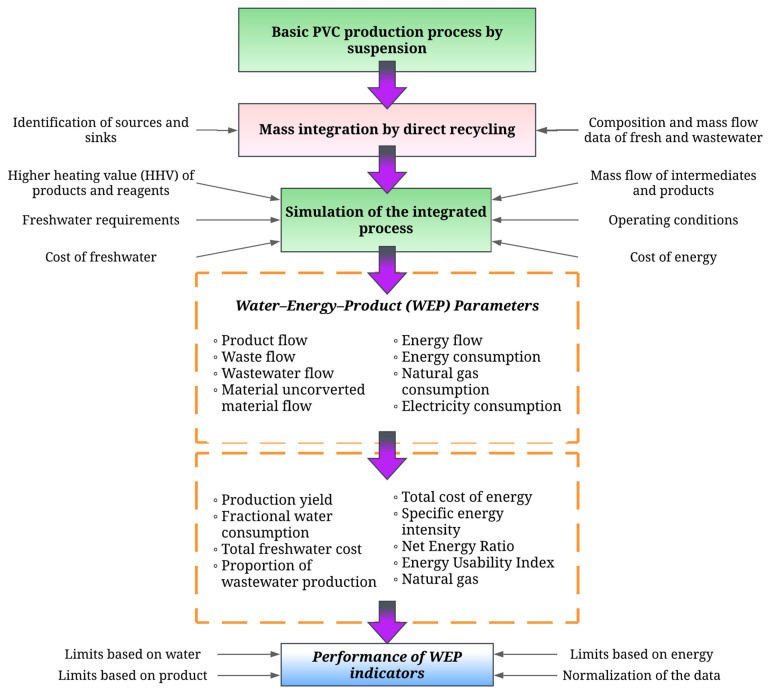
Systematic method for the technical evaluation of the PVC suspension production process with direct water recycling.

Figure 1 shows the methodology used for the water integration with direct recycling of the PVC production process by suspension, based on the approach described by Moreno et al., which combines water integration with a process analysis method using simulation software [24]. The base process of PVC production by suspension on an industrial scale is water-integrated using the graphical method of sink–source mapping. This requires the identification of the different sinks and sources, together with the water flow data and composition of each relevant stream of each one. With the recycling network obtained, a simulation of the integrated process is constructed using AspenPlus V14 software. Thermodynamic models, models for each unit, and operating parameters, among others, are taken into account for its construction. With the extended mass and energy balances provided by the simulator, the technical indicators are calculated to diagnose the process using the WEP methodology developed by the authors [14]. This is used to diagnose the performance of the process under technical parameters in the efficient use of resources.

### 2.1. Process Description

**Figure 2:**
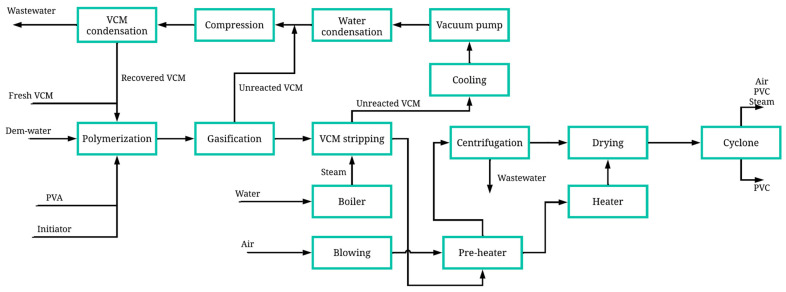
Block diagram of PVC production with energy integration.

Figure 2 details the PVC polymerization process, which is divided into different stages. The polymerization stage involves the use of several batch reactors arranged in parallel, operating at a temperature of 70 °C and a pressure of 10 kg/cm^2^. The reactor feed consists first of freshwater entering at a temperature of 85 °C and a pressure of 3.50 kg/cm^2^. The monomer feed contains both fresh and recirculated vinyl chloride monomers (VCMs), which enter the reactor at 32 °C and 5 kg/cm^2^. The process uses 1152 tons per day of vinyl chloride monomer as raw material. Additionally, polyvinyl alcohol at 20% is added as a stabilizer and 3-hydroxy-1,1-dimethylbutane-2-ethyl-2-methylheptane peroxide at 20% as an initiator, both entering at 32 °C and 10 kg/cm^2^ [25]. PVC production is carried out via the suspension polymerization method in water, where the initiator facilitates the bonding of vinyl chloride monomer particles, forming the polymer, in conjunction with the stabilizer to control the reaction. The reaction is maintained at a constant temperature. When the reaction occurs, a heterogeneous mixture known as slurry is formed, which contains suspended PVC, unreacted VCM, water, and the initiator.

In the gasification stage, the slurry is sent to a gasifier operating at 70 °C and 1.81 kg/cm^2^, which is fed with steam from a boiler that releases steam at 225 °C and 14 kg/cm^2^. The boiler is supplied with freshwater. In the gasifier, due to the pressure change, the unreacted VCM in the mixture separates from the liquid, generating two streams: VCM in a gaseous state and PVC in a liquid state. At this point, the process splits into two streams: monomer recovery and polymer drying.

The gaseous VCM stream moves to the recovery stage, passing through a series of condensers at 50 °C and 1.01 kg/cm^2^ and compressors, where the monomer condenses. Additionally, this stage removes the water present in the VCM stream, achieving VCM recovery for later recirculation. The PVC stream exiting the gasifier contains PVA, the initiator, and excess water. First, it enters a cooling sub-stage, where heat exchange occurs through a heat exchanger, which is supplied with part of the heat from the PVC stream and an air stream passing through a blower. The PVC stream is cooled to 74 °C, while the heat exchanger releases an air stream heated to 91 °C.

The cooled PVC stream then enters a centrifuge to separate the polymer from other compounds (water, PVA, initiator). The centrifuge operates at 1800 rpm, removing 75% of the water present in the slurry, as well as removing PVA and the initiator. The centrifugation product is a wet paste, which must undergo drying. The wet PVC moves to the drying stage.

A dryer operating at 250 °C is fed with air from the heat exchanger, generating a stream of PVC particles with a small percentage of water, the initiator, PVA, and air. To extract pure PVC particles, the stream is passed through a cyclone operating at 1.03 kg/cm^2^. From the top, steam, water, PVA, the initiator, and air are released; from the bottom, granulated PVC exits with 0.01% water, producing a total of 1150.28 tons per day of this polymer.

### 2.2. Water Integration Through Direct Recycling

In this work, the graphical method of the source–sink map developed by El-Halwagi [26] is employed. This map is a visual tool that helps identify recycling opportunities. Initially, the sources of wastewater and potential sinks of the process are identified, which correspond to units consuming freshwater. Next, information is gathered regarding the flow and mass composition of the wastewater generated in the sources and the freshwater used in the sinks. However, before this collection, the critical pollutant (a single contaminant) that conditions or limits the reuse of wastewater in each sink is identified.

The diagram is constructed by plotting the mass flow against the composition of the limiting contaminant. The composition is represented on the *x*-axis, and the flow on the *y*-axis. Each source and sink is assigned a name and a number (Sr for sources and Sk for sinks), based on the amount of the limiting contaminant they contain, ordered from least to most.

To link the streams in the diagram, the “lever-arm” rule is applied. This rule determines how to prioritize the different sources and sinks for implementing the recycling system, as well as guiding the formation of mixed streams (residual and fresh) that maximize recycling through dilution. In each sink, the highest permissible composition of the contaminant is used, and the minimum flow necessary is applied (if a minimum flow exists; otherwise, the maximum flow is used).

The order of minimization of the sources follows specific prioritization rules described by El-Halwagi. These rules establish that the process should be carried out in an ascending manner, starting with the source that has the smallest fresh arm. This procedure continues until the source is completely recycled or until the sink’s limitations prevent further recycling. If a source is exhausted before fully meeting the sink’s needs, the next closest available source is used. This process is repeated with all the sources until they are fully minimized.

To calculate the freshwater requirement of a specific sink, Equation (1) is used:

(1) $$\dot{\left(M_{fresh}\right)}=\dot{\left(M_{z}\right)}*\frac{y_{i}-z_{max}}{y_{i}-z_{min}\;}$$

where $y_{i}$ is the key contaminant composition of a source, $z_{min}$ and $z_{max}$ are the minimum and maximum compositions of the analyzed sink, and $\dot{\left(M_{z}\right)}$ is the maximum flow allowed by this sink. The amount of reused material is calculated by subtracting the sink’s maximum flow from the fresh resource flow obtained, as shown in Equation (2):

(2) $$\dot{\left(M_{r}\right)}=\dot{\left(M_{z}\right)}-\dot{\left(M_{fresh}\right)}$$

This reused flow simultaneously represents the flow that is reduced in the waste streams. If there is residual flow after being linked, it is calculated by subtracting the reused flow.

(3) $$\dot{\left(G_{w}\right)}=\dot{\left(G_{i}\right)}-\dot{\left(M_{r}\right)}$$

### 2.3. Process Simulation of the Energy- and Water-Integrated Case

For the simulation of the integrated process, Aspen Plus V14 software was used. This diagram was constructed based on the authors’ previous work [14] and information from the literature. For the recycling network, the calculations from the integration procedure were used as input data. Additionally, for the convergence of the flowsheet, the Broyden method was employed for the convergence of the units and the recycled streams. A 100% recycling rate was considered, meaning all of the flow is returned to the process, and the mixing and bifurcation of streams were carried out in a standard mixer model.

### 2.4. WEP Technical Evaluation of the Energy- and Water-Integrated Process

Aguilar-Vásquez et al. developed an analysis model to evaluate different aspects of the industrial process from the perspective of water consumption, energy utilization, and PVC production performance, known as the WEP (water–energy–product) method. This method proposes combining process simulation with 11 technical indicators that encompass the efficient use of resources such as water, energy, and unconverted materials, including final production yield, fractional water consumption, waste generation, and others. These aspects provide insights into analyzing the performance of the process, which is energy- and water-integrated with direct recycling. Table 1 provides a detailed description of each indicator.

Based on these WEP indicators, intervals must be established to normalize them and estimate the process performance. This is achieved by determining the best and worst cases as the upper and lower intervals, respectively, as detailed in Table 2. Reference data are taken directly from the literature, primarily focusing on cases similar to the studied process, specifically the suspension PVC production process.

## 3. Results

### 3.1. Water Integration of the PVC Production Process with Direct Recycling

Table 3 presents a summary of the sources and sinks identified in the industrial-scale suspension PVC process. The streams selected include the condensers from the MVC recovery zone (Sr1 and Sr2) and the centrifuge water stream (Sr3). These identified sources are used as transfer agents with fixed characteristics (load and flow) [27]. The Sr1 and Sr2 recovery zones are considered from the recovery of VCM, specifically in condensations carried out for monomer purification; the Sr3 recovery zone arises from the PVC purification stage, following the implementation of centrifugation. The main sinks were the reactor, which represents the primary water consumption in the process (Sk1), and the boiler for steam generation for the stripping column (Sk2). The maximum constraints of these sinks were identified based on the information provided by Blanco and collaborators [28]. PVA was identified as the key contaminant in the process, representing the main limitation for the reuse of process effluents. Due to its properties, PVA has a strong affinity for water, tending to remain in the liquid phase rather than the solid phase [29].

**Figure 3:**
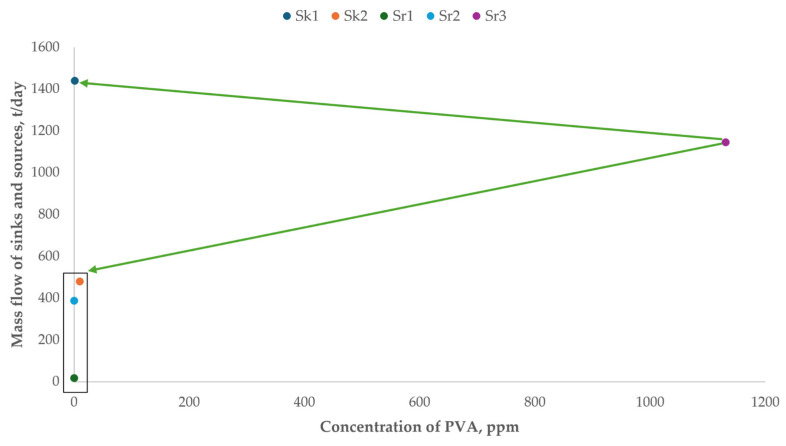
Diagram of sources and sinks of the PVC production process towards direct recycling.

Figure 3 shows the map of sources (Sr1, Sr2, Sr3) and sinks (Sk1, Sk2) identified in the suspension PVC process for water integration with direct recycling. The PVA composition was worked in ppm for each stream. Equations (1)–(3) were used to design the process-water-recycling network, following the rules described in the previous section.

Only the absolute arms of Source 3 with respect to the sinks were considered. This was not performed for Sources 1 and 2, as they are not limited by their composition (traces of PVA). In this figure, it can be seen that Sr1 had a smaller arm for sink Sk2 than for sink Sk1. Therefore, Sr1 was initially linked to Sk2 until its maximum limit was reached (considering the maximum flow of the unit). Then, Sr3 was linked to Sk1 with the same goal. The remaining part of this stream becomes wastewater.

**Figure 4:**
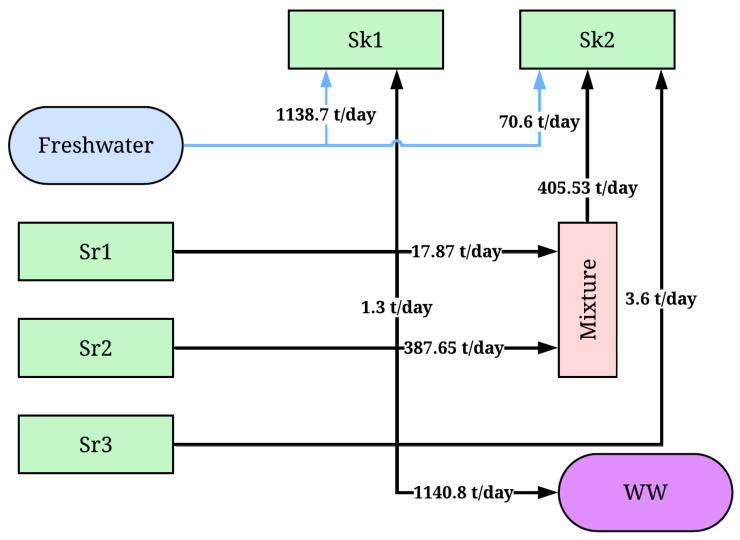
Water-recycling network of the industrial-scale PVC suspension process. Sr1, Sr2, and Sr3 are sources from condensers (Sr1, Sr2) and the centrifuge (Sr3). Sk1 and Sk2 are sinks in the reactor (Sk1) and boiler (Sk2). The mixture is a union of water streams. WW is wastewater.

Figure 4 shows the water integration network for the industrial-scale suspension PVC process. It can be seen that for sources Sr1 and Sr2 (405.5 tons per day), both were directly linked to the boiler (Sk2), and both sources were fully minimized due to their null PVA content. For source Sr3, it was linked according to the maximum limits allowed for the sinks, recycling 1.3 and 3.82 tons/day for sinks Sk1 and Sk2, respectively. This recycling is quite limited because the composition of the mixture of streams from different sources exceeds the maximum allowable contaminant limit for both sinks. This limitation opens up the possibility of exploring effluent regeneration systems. The final freshwater for the reactor was 1138.7 tons/day, while for the boiler, it was 70.6 tons/day. On the other hand, 1140.8 tons/day of wastewater (WW) from source Sr3 was discarded, which was sent to an effluent treatment system for subsequent discharge. A significant limitation for this system is the lack of sinks related to industrial services. Normally, these additional sinks do not have any significant limitations in composition or flow of the stream. If the process sink system is expanded, there is a possibility that the effluents (source Sr3) from the process could be utilized almost to their maximum.

### 3.2. Simulation of the Water-Integrated Suspension PVC Production Process with Direct Recycling

**Figure 5:**
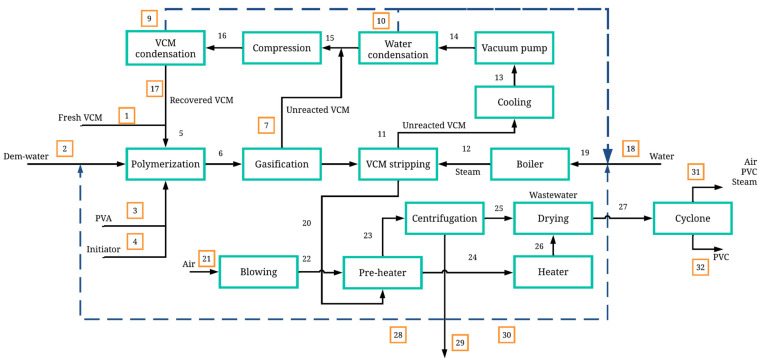
Block diagram of the water and energy integrated suspension PVC production process with direct water recycling.

Figure 5 describes the MVC polymerization process, implementing water integration with direct recycling from the direct-recycling network. For a raw material feed of 1152 tons/day of MVC, polymerization is carried out in a set of parallel batch reactors. Through the water recirculation network, a recirculated water stream (Stream 28) taken from the PVC drying stage with a flow of 2236.26 tons/day is added to the freshwater stream (Stream 2) with a flow of 1438.70 tons/day.

The monomer feed contains fresh MVC (Stream 1) and recirculated MVC (Stream 17). A 20% polyvinyl alcohol stream (Stream 3) is introduced as a stabilizer, and a 20% 3-hydroxy-1,1-dimethylbutane-2-ethyl-2-methylheptane peroxide stream (Stream 4) is used as an initiator. After the polymerization reaction, a slurry is formed containing suspended PVC, unreacted MVC, water, and the initiator.

The slurry is sent to a gasifier, which operates with steam generated by a boiler. The boiler is supplied with freshwater (Stream 18) and two recirculated water streams taken from the PVC drying stage (Stream 30) and MVC recovery stage (Streams 9 and 10). In this unit, unreacted MVC is separated from the liquid, generating a gaseous MVC stream (Stream 7) and a liquid PVC stream (Stream 8).

The gaseous MVC stream is recovered through a series of condensers and compressors, where the monomer is condensed. The liquid phase of the MVC stream has its water content removed, sending the monomer to the polymerization stage (Stream 17), while the residual water is recirculated to the boiler feed (Streams 9 and 10). The liquid PVC stream contains PVA, the initiator, and excess water (Stream 20). First, it is cooled using a heat exchanger, which is fed by some of the heat from the PVC stream, while air enters via a blower (Stream 21). The PVC stream is cooled to 74 °C, while the air exiting the exchanger is heated to 91 °C. The PVC stream is then sent to a centrifuge to separate the polymer from the other components. The equipment operates at 1800 rpm, removing 75% of the water and removing the PVA and the initiator.

The wet PVC moves to the drying stage. A dryer is fed with air from the heat exchanger, producing a stream of particulate PVC with a small amount of water, the initiator, PVA, and air (Stream 27). The pure particulate PVC is separated from the other compounds by a cyclone, which operates at 1.03 kg/cm^2^. The top releases steam, PVA, the initiator, and air (Stream 31), while the bottom exits with granulated PVC containing 0.01% water (Stream 32), producing 1150.28 tons/day of PVC.

The water exiting the centrifuge is sent to a cooler, where the small amount of PVC, PVA, and the initiator present in the water is released (Stream 29). The removed water is recirculated to the polymerization stage (Stream 28) and the boiler feed (Stream 30). Through water integration, these streams help reduce water consumption.

**Figure 6:**
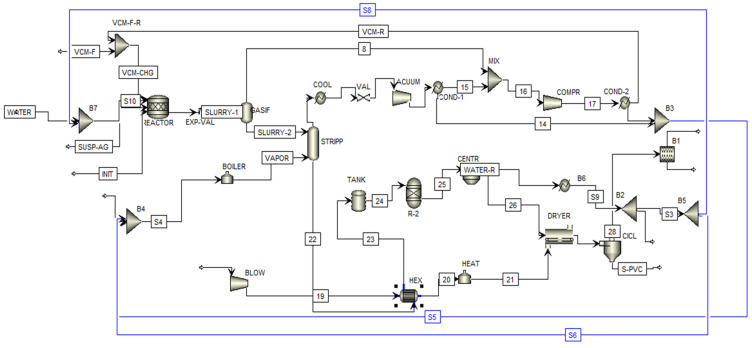
Diagram of the simulation of the water-integrated suspension PVC production with direct recycling.

Figure 6 shows the simulation of the integrated process using a direct water recirculation network. Streams S8 and S6 are directed to the reactor for polymerization and to the boiler for separating unreacted MVC from the slurry. These units correspond to the highest freshwater consumption. The other side of the simulation involves the PVC production with direct recirculation, highlighting the sources of wastewater. Wastewater streams were identified in the MVC and PVC purification stages.

The water-recycling network was incorporated into the simulation. The streams initially considered wastewater, shown as residue in the purification stages, and were redirected to the reaction stage and the boiler, reducing the wastewater to 1144.1 tons/day in the simulation, in line with the presented network. Additionally, an air separation stage was included to separate excess air generated during PVC purification from the wastewater, with the objective of sending this excess air to an effluent treatment system for subsequent discharge.

### 3.3. Technical Evaluation of the Water-Integrated Suspension PVC Production with Direct Recycling Using WEP Indicators

Table 4 presents the flow data related to the inputs of raw materials, water, and energy used in the various stages of the process, including separation and suspension operations, as well as the polymerization reaction. The corresponding outputs, such as wastewater and the final product, are also included. These parameters are part of the WEP indicators, which are essential for technical analysis. Their identification is crucial as they are directly linked to resource management. Additionally, quantifying the consumption of freshwater and energy needed to produce a certain amount of product allows for evaluating the overall efficiency of the process.

Table 5 contains the results obtained for each WEP indicator present in the suspension PVC production process. To determine if the raw material was well utilized, indicators such as production yield and the Index of Unconverted Material Reused (IRUM) were used. For the former, a 99.8% yield was achieved, which is high and is mainly due to the high conversion and subsequent recycling of unconverted VCM in the reaction stage of the process. This demonstrates the maximum utilization of raw materials, as the result of this indicator shows that most of the raw material used for PVC production was transformed and is reflected in the mass flow of the obtained product.

In this sense, it is clear that the IRUM, which is directly applied to the VCM for practical reasons and to allow for better analysis of its use within the production process, was 100%. This result clearly shows that all raw material that does not react is being recirculated into the process. This mainly occurs in the VCM recovery stage, with minimal amounts still present in the residual water, which are reintroduced into the process due to the direct recycling integration, further minimizing potential raw material losses and allowing for optimal use.

Because the freshwater consumption is not directly counted as raw material in the previous indicator, despite being the medium in which the reaction occurs and thus an indispensable factor, there are other indicators such as the fractional water consumption (FWC) and freshwater cost (TCF) that allow us to describe the process performance in the efficient use of this resource.

From Table 4, we find that the FWC indicator is 2.2 m^3^/t, noting that this result was calculated not only considering the amount of demineralized water used in the process but also the total amount of freshwater. With the reference that the average water consumption for polymer production is 3 m^3^/t of PVC according to the BREF [30], we can see that there is a lower use of potable water for PVC production with direct wastewater recycling. This is due to a lower need within the production process by reusing water that would otherwise be discarded, now being utilized, generating less consumption of potable water, which allows for better use of this resource in the process. This should be reflected in a lower freshwater cost, as clearly, lower consumption benefits the associated costs of this resource within the process. Thus, the performance of the total freshwater cost indicator is 29.4%, while the result obtained for a PVC production process without water integration was only 8.3%, according to the study by Aguilar-Vasquez et al. [14]. We can see that there is a noticeable improvement in the costs associated with the supply of potable water when direct wastewater recycling is implemented, showing a performance superior to the regular case, being more than three times more efficient.

Another indicator that is crucial both for the sustainability of the process and for the costs related to the water resource is the proportion of wastewater production (WPR), which is 45%. This means that the flow of wastewater leaving the process is less than half of the potable water entering it. In general terms, the suspension polymerization process requires a large amount of water, primarily because it serves as the medium in which the MVC monomer breaks down, producing the polymer chains that are the target production, in this case, PVC. This process, without water-recycling streams, would generate considerable amounts of wastewater, which would affect the performance and economy of the process due to higher costs related to freshwater consumption and wastewater treatment.

The WPR indicator, along with the previously mentioned ones, demonstrates the benefits generated by recycling wastewater back into the process. This implementation becomes an essential factor in industrial water management, as presented in the study by Pietro et al. [31]. This, in turn, directly influences other indicators, such as FWC and TCF. In this sense, and precisely because analyzing the use of potable water in the production process is one of the objectives of this study, it is necessary to address several points related to this issue. Firstly, it must be noted that the production of wastewater within the process is significant due to the conditions of the same, implying higher treatment costs. It is also necessary to thoroughly address the topic of process sustainability, as it is undeniable that over time, there has been an increasing societal concern about the lack of water. If an integrated and sustainable water management approach is not ensured, the growing demand for water will have severe consequences for the environment. Therefore, industries need to seek alternatives to be more independent from the water supply for their production processes, as argued in the study by Pietro et al. [31], especially in a process like PVC production, which, as previously mentioned, is one of the most important polymers today. A solution that reduces costs in the potable water service without affecting the product quality is to recycle wastewater back into the process, as is the case here. For a PVC production process without direct wastewater recycling, the mass flow of wastewater would be 1552 m^3^/day, as presented in the study by Aguilar et al. [14]. However, our results show that the implementation of water integration reduced the production of wastewater to 1144 m^3^/day.

With a value of 3.4 GJ per ton, the indicator for the energy-specific intensity (ESI) is considerably lower compared to other PVC production processes that exceed 5 GJ per ton, such as the one obtained by Franklin Associates with 5.1 GJ per ton [32]. While this is an encouraging result, it is important to recognize that the aforementioned process includes additional equipment necessary for the proper functioning of the plant, such as pumping systems, control systems, and others, which would have made a considerable contribution if they had been included in the analysis.

Since the process primarily uses natural gas for energy consumption, with 52.5 m^3^ of natural gas and 1.6 kWh required per ton of PVC, it is clear that the process uses minimal amounts of electricity, which is used for equipment such as blowers, dryers, centrifuges, and others. Meanwhile, natural gas is used to power equipment with higher energy demands, such as boilers, burners, and flash units. Annually, the cost of energy, including both electricity and natural gas, amounted to USD 12,707,134, as shown in Table 5.

Regarding the energy usability index (EUI) and the net energy ratio (NER), values of 5.4 and 0.9 were obtained, respectively. The net energy ratio is mainly used to show whether the product in question is viable for use as a biofuel, as this indicator demonstrates that when the value is greater than one, the product contains more energy associated with it than the energy used for its production. This allows us to say that PVC, despite not being commonly used for this purpose due to the production of chlorinated products and dioxins, which cause significant human health concerns according to Park et al. [33], has notable potential to be used as fuel based on the value obtained in this study. Recently, various studies have demonstrated that the combustion of mixtures containing a large amount of PVC can maximize its potential through controlled combustion and safely and effectively fractionate all the toxic compounds generated [34].

**Figure 7:**
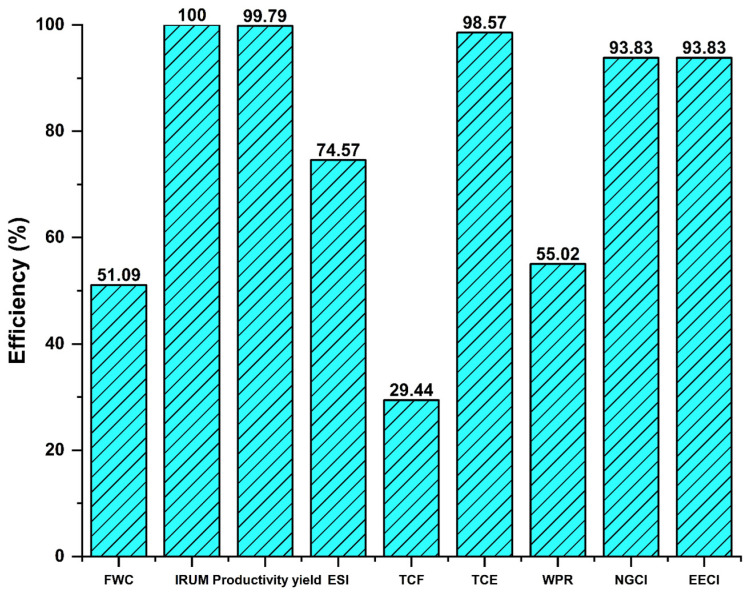
Performance of technical indicators for the suspension PVC production process with direct water recycling.

Figure 7 shows the performance of the studied indicators related to water, energy, and product in the suspension PVC production process. Except for the NER and EUI, which, due to their nature and calculation methodology, cannot be normalized in percentage terms because they do not have a best- and worst-case interval, the figure illustrates that the production yield and the index of unreacted material reused indicators exhibit the best efficiencies, with approximately 100%, indicating excellent utilization of the raw material in relation to the amount of product being obtained.

In the same context, the indicators related to water management show significant improvement, which can be attributed to the water integration with direct recycling. This integration reduced both potable water consumption and wastewater production without affecting the quality or quantity of the product obtained, directly benefiting the process economy by reducing the total freshwater cost by approximately 16%, from 693,080 USD/year according to the results obtained by Aguilar et al. [14] to 584,285 USD/year. There was even an improvement in this aspect, as the recycling of wastewater also allowed almost total reuse of unreacted raw material.

The indicators associated with energy showed considerable improvements. Due to the prior energy integration, natural gas consumption decreased to 6.9 m^3^ per ton of PVC produced. As mentioned earlier, the process primarily used natural gas for plant operations, which is positive, and adding to this, the integrated process requires less consumption, with these improvements reflected in lower energy-related costs. However, for water integration with direct recycling, additional equipment is required for proper functioning, significantly increasing electricity consumption, but this is not a negative alarming factor, as the process still performs better compared to a process that is not energy- or water-integrated.

## 4. Conclusions

Through this work, the WEP analysis methodology was proposed to conduct the technical evaluation of the suspension PVC production process by implementing direct recycling streams of wastewater. Through this method, various indicators related to water, energy, and product management were evaluated, hence the acronym WEP (water–energy–product). The indicators that allow us to visualize the process performance in terms of water management are FWC and WPR, which show improved handling and utilization of this resource, demonstrating a performance of 51.1% and 55.0%, respectively, increasing compared to the process without integration, which was 41% and 54%. This suggests that water integration allowed the process to become more efficient in the use of potable water, reducing not only the required quantity and associated cost but also the production of wastewater, which would generate an additional treatment cost. The freshwater cost results were 584,285 USD/year, a value significantly lower than those reported in the literature studies on PVC production. The unreacted material reuse index shows a performance of 100%, partially due to the recycling of wastewater, demonstrating that the majority of MVC that initially did not react is being used, thus benefiting the production yield with a performance of 99.8%. The TCE, NGCI, and EECI indicators show optimal utilization due to the use of natural gas as the primary energy source and energy integration. However, the energy intensity (ESI) indicator is high due to the implementation of heat transfer equipment to control water recirculation, showing a consumption of 3.59 MJ/t for PVC production. From the results, it is observed that by direct water recycling, better water management is achieved, reducing the consumption of freshwater while positively contributing to the cost of this resource. However, although energy integration improved process efficiency, the inclusion of new equipment as part of water integration resulted in higher electricity consumption. Therefore, it would be advisable to implement additional techniques to optimize this new energy expenditure without affecting other parts of the process. It is noteworthy that without prior energy integration, energy-related costs would have been much higher than in the case with no integration.

Based on this work, we can conclude that the WEP method and its indicators show a significant improvement in the suspension PVC production process with water integration and direct recycling, both energetically and from a water management and consumption perspective, without affecting PVC production. In view of the positive results obtained, future research could evaluate the cumulative impact of wastewater recycling on the quality of the final product, as well as analyze the potential replicability of the system in other industries with high water consumption. These studies would help to consolidate water recycling as an effective strategy within a responsible production framework.

## Figures and Tables

**Table 1:** Description of indicators for technical process analysis.

Variable	Units	Equation
Production yield	%	$\gamma_{i}=\frac{mass\;flow\;of\;product}{mass\;flow\;of\;main\;feedstock}\times 100\%$
Fractional water consumption (FWC)	m^3^/t	$FWC=\frac{volume\;flow\;of\;freshwater}{mass\;flow\;of\;product}$
Total cost of freshwater (TCF)	USD/day	TCF=flowrate of freshwater consumed×cost of freshwater
Wastewater production ratio (WPR)	%	$WPR=\frac{wastewater\;volumetric\;flow\;}{freshwater\;volumetric\;flow}\times 100\%$
Index of reused unconverted material (IRUM)	%	$IRUM=\frac{reused\;material\;i\;mass\;flow}{unconverted\;material\;i\;mass\;flow}\times 100\%$
Total cost of energy (TCE)	USD/day	TCE=total energy consumed×cost of energy
Energy-specific intensity (ESI)	MJ/t	$R_{ESI}=\frac{total\;energy\;consumed\;}{product\;mass\;flow}$
Net energy ratio (NER)	Dimensionless	$NER=\frac{\;product\;calorific\;power\times product\;mass\;flow}{total\;consumed\;energy+(feedstock\;calorific\;power\times feedstock\;mass\;flow)}$
Energy usability index (EUI)	Dimensionless	$EUI=\frac{product\;calorific\;power\times product\;mass\;flow}{total\;consumed\;energy}$
Natural gas consumption index (NGCI)	m^3^/t	$NGCI=\frac{total\;natural\;gas\;consumed}{product\;mass\;flow}$
Electric energy consumption index (EECI)	kWh/t	$EECI=\frac{total\;electricity\;consumed}{product\;mass\;flow}$

**Table 2:** Description of WEP indicators with their respective reference values.

Variable	Worst Case	Best Case
Production yield	0%	100%
Fractional water consumption (FWC)	4${\;m}^{3}/t$	0.5${\;m}^{3}/t$
Total freshwater cost (TCF)	2514 USD/day	193 USD/day
Wastewater production ratio (WPR)	100%	0%
Index of reused unconverted material (IRUM)	0%	100%
Total cost of energy (TCE)	0.41 USD/kWh (100% of the energy used comes from electricity)	10 USD/MMBTU (100% of the energy used comes from natural gas)
Energy-specific intensity (ESI)	5000 MJ/t	3500 MJ/t
Natural gas consumption index (NGCI)	0%	100% of the energy entering the process
Electric energy consumption index (EECI)	100%	0% of the energy required

**Table 3:** Consolidation of sources and sinks of the studied process.

Source	Mass Flow [t/Day]	PVA [mg × L^−1^]	Mass Fraction	Load [t/Day]
Sr1	17.87	17,923.77	0	0
Sr2	387.659	388,825.47	0	0
Sr3	1145.92	1,145,506.49	1131.37	1296
**Source**	**Mass Flow [t/Day]**	**PVA [mg × L^−1^]**	**Mass Fraction**	**Load [t/Day]**
Sk1	1440	1	1.00301 × 10^−6^	0.0014
Sk2	480	10	1.00301 × 10^−5^	0.0048

**Table 4:** Process parameters used in the WEP analysis present in the water and energy suspension PVC production process.

Parameter	Units	Description	Value
Mass flow of raw material (VCM)	t/day	total flow of VCM entering the process	1152
Total mass feed flow	t/day	total flow of substances entering the process, including water, reactants, etc.	9003
Mass flow of recycled raw material	t/day	total flow of VCM being recycled to the process	288
Mass flow of product	t/day	total flow of PVC leaving the process	1150
Total volumetric flow of water	$m^{3}$/day	volume of freshwater used in the process	1623
Total volumetric flow of wastewater	$m^{3}$/day	volume of wastewater used in the process	1144
Total energy consumed	GJ/day	total energy used during the process (includes cooling and heating)	3861.8

**Table 5:** Results of the WEP indicators.

Indicators	Units	Value
Production yield	%	99.8
Fractional water consumption (FWC)	$m^{3}/t$	2.2
Total freshwater cost (TCF)	USD/year	584,285
Wastewater production ratio (WPR)	%	45
Index of reused unconverted material (IRUM)	%	100
Total cost of energy (TCE)	USD/year	12,707,134
Energy-specific intensity (ESI)	MJ/t	3359
Natural gas consumption index (NGCI)	$m^{3}/t$	52.5
Electric energy consumption index (EECI)	kWh/t	1.6
Net energy ratio (NER)	-	0.9
Energy usability index (EUI)	-	5.4

## Data Availability

The data that support the findings of this study are available from the corresponding author, Á.D.G.-D., upon reasonable request.

## Abbreviations

VCM	vinyl monochloride
PVC	polyvinyl chloride
WEP	water–energy–product
FWC	fractional water consumption
TCF	total freshwater cost
WPR	wastewater production ratio
IRUM	unreacted material reuse index
TCE	total energy cost
ESI	energy-specific intensity
NGCI	natural gas consumption index
EECI	electricity energy consumption index
NER	net energy ratio
EUI	energy usability index
